# Establishment and Validation of an Interferon-Stimulated Genes (ISGs) Prognostic Signature in Pan-cancer Patients: A Multicenter, Real-world Study

**DOI:** 10.7150/ijbs.71385

**Published:** 2022-05-21

**Authors:** Zheng Zhou, Yujia Zheng, Shaobo Mo, Shuofeng Li, Xinlei Zheng, Ran Wei, Tao Fan, Tianli Chen, Chu Xiao, Chunxiang Li, Jie He

**Affiliations:** 1 Department of Thoracic Surgery, National Cancer Center/National Clinical Research Center for Cancer/Cancer Hospital, Chinese Academy of Medical Sciences and Peking Union Medical College, Beijing, China.; 2 Department of Colorectal Surgery, Fudan University Shanghai Cancer Center, Shanghai 200032, China.; 3 Department of Colorectal Surgery, National Cancer Center/National Clinical Research Center for Cancer/Cancer Hospital, Chinese Academy of Medical Sciences and Peking Union Medical College, Beijing 100021, China.; 4 Department of Pharmacology, Nanjing University of Chinese Medicine, Nanjing, Jiangsu, 210023, China.

**Keywords:** Natural Killer Cells, Tumor Biomarkers, Interferon-stimulated Genes, Pan-cancer Study

## Abstract

Our study aims at developing an interferon-stimulated genes (ISGs) signature that could predict overall survival (OS) in cancer patients, which enrolled a total of 5643 pan-cancer patients. Linear models for microarray data method analysis were conducted to identify the differentially expressed prognostic genes in the global ISGs family. Time-dependent receiver operating characteristic (ROC) and Kaplan-Meier survival analysis were used to test the efficiency of a multi-gene signature in predicting the prognosis of pan-cancer patients. The prognostic performance and potential biological function of gene signature were verified by quantitative real-time PCR in a pan-cancer independent cohort. Three ISGs genes were finally identified to build a classifier, a specific risk score formula, with which patients were classified into the low- or high-risk groups. Time-dependent ROC analyses proved prognostic accuracy. Then, its prognostic value was validated in seven external validation series. A nomogram was constructed to guide the individualized treatment of patients with lung adenocarcinoma. Biological pathway and tumor immune infiltration analysis showed that the signature might cause poor prognosis by blocking NK cell activation. Finally, the signature in our centers was confirmed by real-time quantitative PCR. A robust ISGs-related feature was discovered to effectively classify pan-cancer patients into subgroups with different OS.

## Introduction

According to the latest worldwide cancer statistics, breast cancer (BRCA), lung cancer, and colorectal cancer (CRC) separately rank the first, two, and three prevalent malignant tumors in the world 1, while lung and colorectal cancer cause the most and the third cancer-related death every year. Lung adenocarcinoma (LUAD) and colon adenocarcinoma (COAD) are the most common subtypes of lung cancer and CRC, respectively. Even with the same pathological subtype, the difference in prognosis between breast cancer, lung cancer, and colorectal cancer still varied significantly due to inter-/intra-tumor heterogeneity 2. For those cancer patients with poor prognosis or even untreated, the introduction of immunotherapy has completely changed the mode and method of cancer treatment and improved the survival rate 3. Kidney clear cell carcinoma (KIRC) stands out as one of the most immune-infiltrated tumors in pan-cancer comparisons, which may be promising to respond efficaciously to immunotherapy 4. However, it is still unclear which patients with poor prognoses should receive immunotherapy to achieve better survival.

Genomic abnormalities were assessed by pan-cancer analysis utilizing sequencing methods, universal model systems and projects, regardless of the tumor origin 5. The data of prevalent pan-cancer studies were mainly from the TCGA database, which stored sequences of all transcripts from more than 30 cancers 6. Molecular similarities appeared in cancers originating from different organs according to the results of pan-cancer analysis, compared to those, which had different genomic profiles, originating from the same tissue 7, 8. Thus, the trend of genomic analysis to classify patients into subtypes was on the basis of pan-cancer data. Pan-cancer prognostic genomic analysis was required to make up for excessive hidden defects in the practical application of the mainstream staging system, which has been determined to have an association with cancer prognosis. Predominant assessment models encompassed too many independent variables and were limited by the scales and tumor types involved in validation cohorts, so it was difficult to warrant that these models can effectively and stably predict and stratify patients^9^. Therefore, this study aimed to stratify patients with fewer variables to obtain higher operability and economic value. Moreover, more and more pan-cancer studies, the key prognostic genes of common cancers, and their impact on tumor biology and microenvironment have not been systematically analyzed, which was also the significance of this research.

The expression of classical interferon-stimulated genes (ISGs) that have key immune effector functions was induced by the IFN receptor and the Janus kinase (JAK)-signal transducer and activator of transcription 1 (STAT1) pathway 10. The type I interferon signaling promoted dendritic cell function and CD8 T cell cross-priming, while type II interferon signaling changed tumors to STAT1-related epigenomic. Both IFNs augmented the expression of ISGs which mediated anti-tumor response 11. Decades of research have proved that many of the protein products encoded by ISGs made contributions to one or more cellular outcomes, including antiviral defense, antiproliferative activities, and stimulation of adaptive immunity^12^. Nevertheless, few have been characterized with respect to anti-tumor potential. Only one study found that the expression of ISG was correlated with radiotherapy resistance and outcome in breast cancer patients 13. More studies were required to know about their anti-tumor activity, their target specificity, and their mechanisms of action^14^.

In our study, we divided TCGA pan-cancer cohorts into subgroups based on the medium of their overall survival (OS). ISGs family was related to immune microenvironment and prognosis. Then we demonstrated that the gene signatures constructed by the three ISGS, NDC80, NPAS2, and AHNAK2, were significantly correlated with the prognosis of patients and seemed better than the traditional staging criteria in TCGA-LUAD cohorts and seven independent validation series. An intuitive and comprehensive nomogram was developed to predict the probability of OS in LUAD patients. Biological pathway and tumor immune infiltration analysis showed that the signature might cause poor prognosis by blocking NK cell activation.

## Materials and Methods

### Publicly available mRNA data and ISGs sets

**Figure 1:**
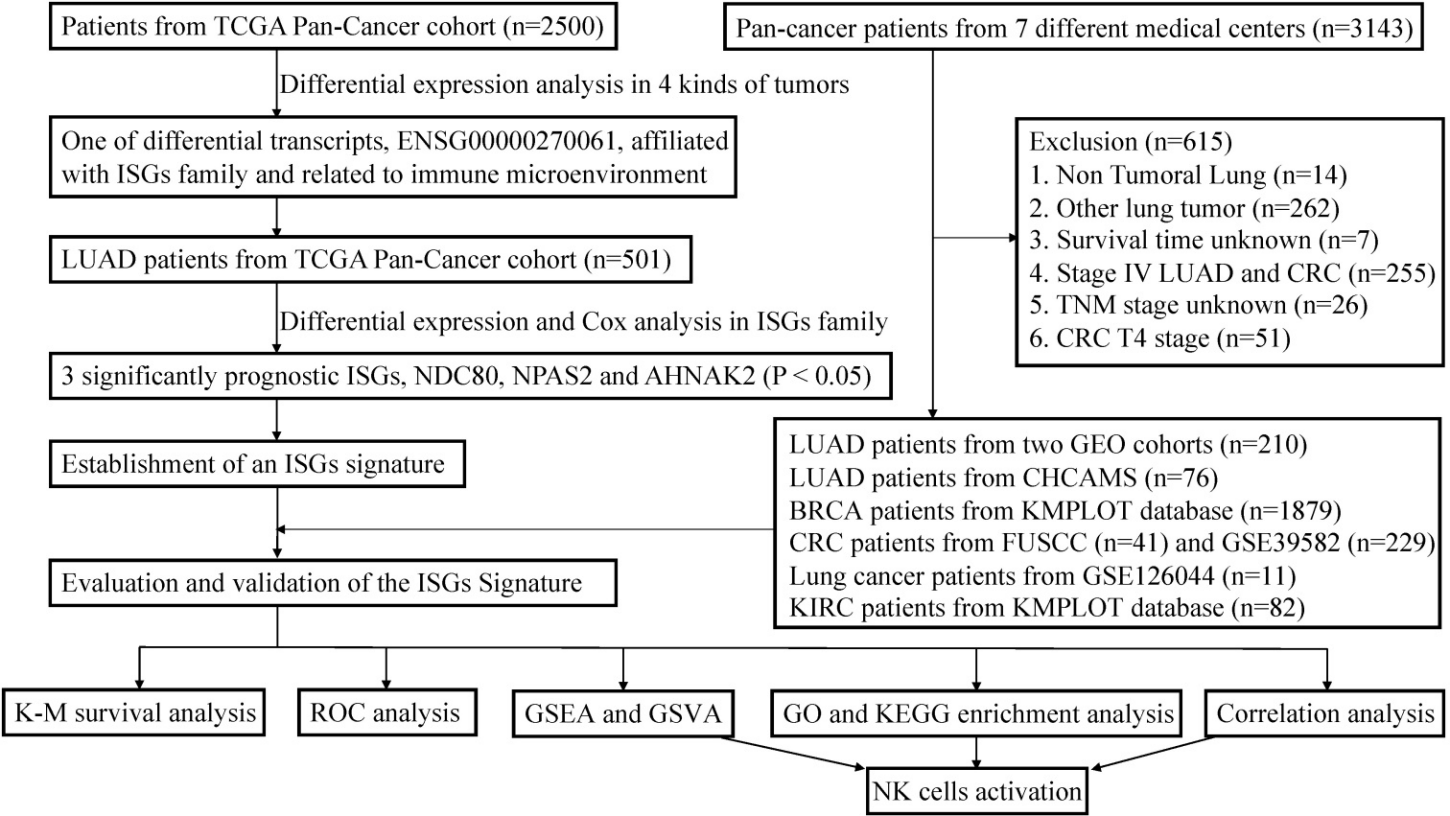
The workflow of establishment and validation of an interferon-stimulated genes (ISGs) prognostic signature in pan-cancer patients.

This study incorporated data from two publicly available datasets. The detailed workflow was shown in Figure 1. TCGA data of samples from pan-cancer patients (Illumina HiSeq 2000) were acquired from the UCSC Xena (https://tcga.xenahubs.net). Using bioinformatic technology, we can use transcriptome data to analyze the immune infiltration and biology characteristics in tumors for pan-cancer cohorts, including LUAD, BRCA, KIRC, and COAD patients 15. More LUAD and COAD cases were selected from GSE30219, GSE50081, GSE126044, and GSE39582. These datasets were downloaded from the GEO database (http://www.ncbi.nlm.nih.gov/geo/), to serve as one of the individual validation sets. More gene expression and prognosis data of BRCA and KIRC patients were downloaded from Kaplan-Meier (K-M) plotter database (http://kmplot.com/analysis/) 16, 17. After log2 transformation and quantile normalization, mRNA expression data detected with more than one probe were calculated by mean expression. ISGs list was excerpted from previous research 14. Since this study paid particular attention to the response of immunotherapy, patients with advanced KIRC and LUAD were specifically included in the validation cohorts to evaluate the predictive performance. Patients with incomplete information were excluded.

### Immune infiltration estimation

To explore the immune infiltration in pan-cancer, CIBERSORT was used to calculate the proportion of 22 immune cells and revealed the detail of immune infiltration 18, 19, while R package “ssGSEA” was associated with supplied cell makers 3.

### Differentially-expressed analysis and signature generation

Linear model for microarray data (LIMMA) method was conducted to evaluate differentially expressed genes (DEGs) between pan-cancer patients who had different OS statuses. The Cox proportional hazards regression model was used to identify significant prognostic genes and to determine correlation estimated coefficients, which were employed to calculate a risk score formula with the expression of optimized genes.

### Statistics for classification, prediction, and validation in the TCGA and GEO series

Patients were divided into high-risk and low-risk groups according to the signature formula. After that, we implemented time-dependent receiver operating characteristic (ROC) analysis to determine the cut-off value and to calculate the area under the curve (AUC) for 1‐, 3‐, and 5‐year OS and RFS in order to confirm the signature performance based on the “survivalROC” R package 20. The K-M survival curve analyses and log-rank tests were used to evaluate the prognostic significance of the risk score formula. Evaluation of the relationship between the distribution of patients' risk scores and survival and recurrence status was conducted. Based on “ComplexHeatmap” R package, the research group constructed a heatmap with cluster analysis in view of the gene expression difference 21. The same protocol was used to verify the signature in GSE30219, GSE50081, and GSE39582 to further investigate the classification constancy. Univariable Cox regression analyses were used to compare the effects of signature and other clinicopathological variables in the training and validation cohorts. The variables with *P* < 0.05 in the univariate model were used to construct a nomogram. ROC and K-M survival analyses were used to evaluate the nomogram.

### Pan-cancer validation series including department of thoracic surgery, National Cancer Center (NCC)/Cancer Hospital, Chinese Academy of Medical Sciences (CHCAMS) and Fudan University Shanghai Cancer Center (FUSCC)

To further substantiate that the results are significant regardless of the data set and tumor type in the study, we verified the results in pan-cancer validation cohorts, which comprised patients from CHCAMS, FUSCC, and K-M plotter database. This study retrospectively analyzed 76 LUAD patients and 41 CRC patients who underwent radical surgery in CHCAMS and FUSCC from 2011 to 2014. The study design was approved by the ethics committee or institutional review board of each participating clinical center, and the written informed consent of all patients was provided before enrollment. According to the manufacturer's protocol, this study conducted total RNA extraction and reverse transcription. The primers used to amplify specific genes are shown in Table S1. Furthermore, this research enrolled 1879 BRCA patients and 82 KIRC patients from the K-M plotter database to compose extensive pan-cancer series. Since KIRC patients benefited significantly from immunotherapy and immunotherapy was only suitable for specific patient groups in the clinic, this study only included stage IV KIRC patients to delve into the potential benefit population of immunotherapy. The same protocol was applied to determine the signature in this pan-cancer validation series.

### Functional enrichment analysis and gene set variation analysis (GSVA)

Functional enrichment analysis of KEGG and GO pathway was performed to determine significantly enriched pathways of DEGs correlated with the signature using the R package “clusterProfiler” 22. Biological pathways with *P* < 0.05 were considered as significant using functional annotation chart options with the whole human genome as background. GSVA was conducted to measure the signaling pathway variation score for each sample in TCGA-LUAD cohorts based on “GSVA” R package 23. Meanwhile, Gene Set Enrichment Analysis (GSEA) was also performed between different risk subgroups via “javaGSEA” to obtain GSEA results 24.

### Statistical analysis

Most analyses used in this study were performed by R software (version 4.1.1). WilcoxTest was used to compare the infiltration of immune cells in two groups. For the survival analysis, *P* value was calculated with the log-rank test. *P* < 0.05 was considered as statistically significant.

## Results

### Correlation of genome and clinical parameters in pan-cancer cohorts and construction of predicting signature

**Figure 2:**
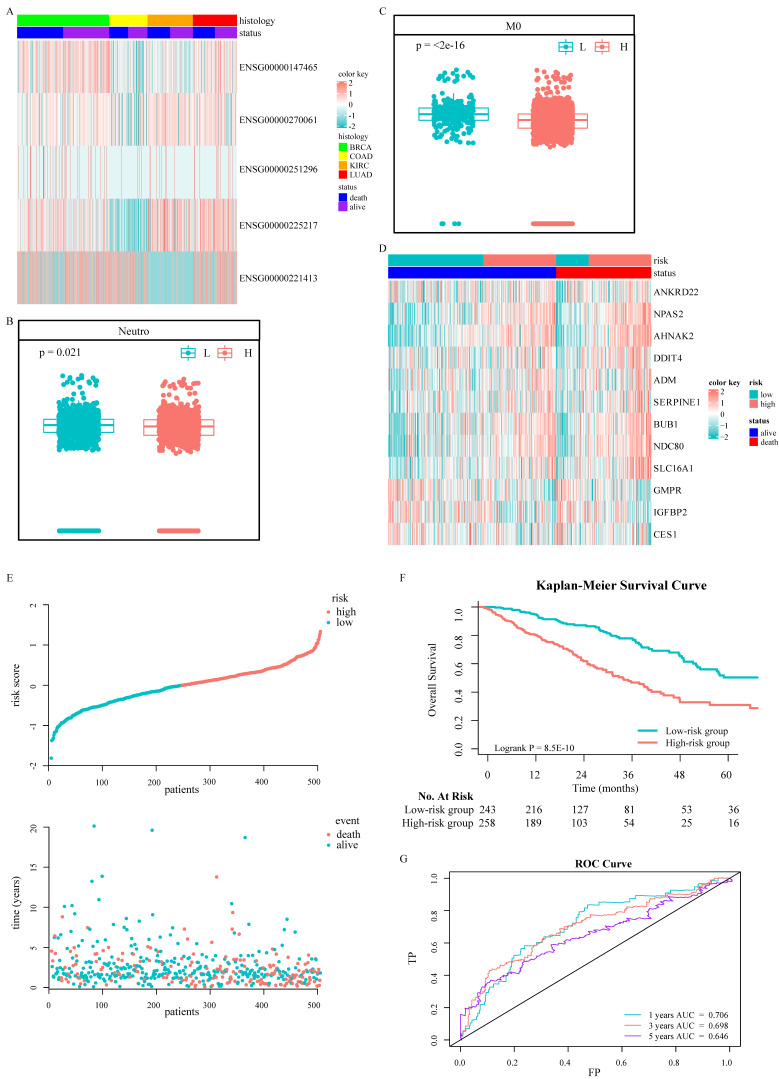
Identification and establishment of ISGs-related signature: Five DEGs in the TCGA pan-cancer cohorts **(**A). The association between ENSG00000270061 and neutrophils infiltrated (B). The association between ENSG00000270061 and non-activated macrophages (C). The prognostic ISGs defined by the LIMMA method in the TCGA-LUAD cohort (D). The distributions of the risk score and survival status of LUAD patients (E). Kaplan-Meier survival curves of OS between high-risk and low-risk patients in the TCGA-LUAD cohort (F). AUC values of ROC predicted 1-, 3- and 5-year OS of the signature in the TCGA-LUAD cohort (G).

A total of 2500 patients were enrolled from TCGA pan-cancer cohorts, including 1045 BRCA patients, 437 COAD patients, 517 KIRC patients, and 501 LUAD patients, with a medium OS of 28 months. Among them, women accounted for 67.36%. The analysis of DEGs suggested that important genes were associated with prognosis (Fig. 2A). Only five differential transcripts in four carcinomas were concerned (Fig. S1A-S1D and Table S2). A transcript, ENSG00000270061, was sense intronic to CCDC92, which was affiliated with the ISGs family. CCDC92 was dramatically deregulated in pan-cancer patients with a worse prognosis (Fig. S2), with a fold change ≥ 1.2. Furthermore, the relationship between the transcription of CCDC92 and the immune microenvironment was detected. The upregulating transcription was associated with lower neutrophils and macrophages infiltration (Fig. 2B and 2C). 315 ISGs were excerpted as ISGs family (Table S3), in which 12 ISGs were found as helpful prognostic genes by the LIMMA method, with a fold change ≥ 1.5 (Fig. 2D and S1E-S1F). A Cox proportional hazards regression model was applied to select the most predictive genes, which identified a final set of 3 genes, including NDC80, NPAS2, and AHNAK2 (*P* < 0.05). Upregulating those ISGs predicted worse survival in LUAD patients. We also calculated a risk value as follows: Risk Value = (0.2445880× NDC80 expression) + (0.1861538 × NPAS2 expression) + (0.1242815× AHNAK2 expression). This formula was used to calculate the risk score for each patient in the TCGA-LUAD, five individual validation cohorts downloaded from public databases and two Chinese medical centers. The demographic and clinical characteristics of the GEO and Chinese cohorts were shown in Table 1, while the K-M plotter database did not provide more clinical information systematically. In the training cohort, the optimized risk value was used in LUAD patients from TCGA set to divide them into high-risk and low-risk groups (Fig. 2E). The K-M survival analyses displayed that the OS in the high-risk group was significantly worse than the low-risk group (Fig. 2F, *P* < 0.0001). The areas under the time-dependent ROC curves were calculated to assess the prognostic performance. The three-ISG-based signature had AUC values of 0.706, 0.698, and 0.646 in TCGA LUAD set at 1, 3, and 5 years, respectively (Fig. 2G).

### Validation of the prognostic model for overall survival in pan-cancer cohorts

**Figure 3:**
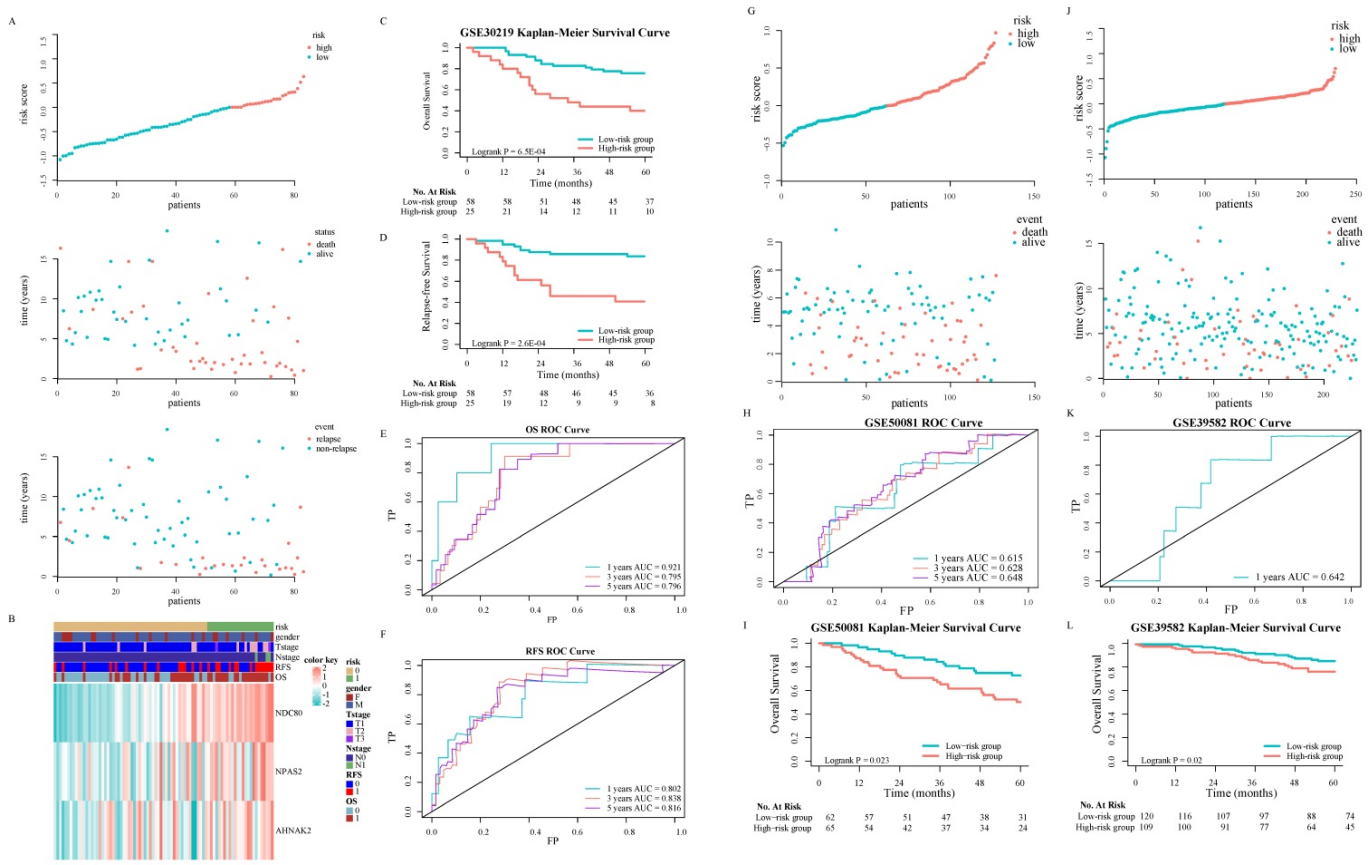
Validation of the 3-ISGs signature in GSE30219, GSE50081 and GSE39582 independent cohorts: The distributions of the risk score and survival status of LUAD and CRC patients (A, G and J). The expression pattern of the 3-ISGs signature and clinical features of patients from GSE30219 (B). Kaplan-Meier survival curves of OS (C, I, and L) and RFS (D) between high-risk and low-risk patients in three independent cohorts. Time-dependent ROC curves at the OS (E, H and K) and RFS (F) time of 1, 3, and 5 years.

GSE30219 was used as a validation cohort to measure the three-ISG-based signature. Patients from GSE30219 were divided into a high-risk group (N = 58) and a low-risk group (N = 25) with the same formula (Fig. 3A). The heatmap showed patients' clinical characteristics and detailed expression patterns of 3 ISGs (Fig. 3B). K-M survival analyses revealed that patients in the low-risk group had significantly better OS and relapse-free survival (RFS) than the high-risk group (Fig. 3C and Fig. 3D). Similarly, time-dependent ROC analyses were conducted to assess the prognostic function of the three-ISG-based classifier, with AUC 0.921, 0.795, and 0.796 at the OS time of 1, 3, and 5 years, respectively (Fig. 3E). For predicting relapse in GSE30219 set at 1, 3, and 5 years, the signature had AUC values of 0.802, 0.838, and 0.816, respectively (Fig. 3F). GSE50081 and GSE39582 were arranged as pan-cancer cohorts after the research group demonstrated that the prognostic model had a robust ability to predict LUAD patients' OS and RFS. Due to tumor heterogeneity, we matched clinicopathological features in two datasets to determine the best applicable population of the predicting model. A total of 229 patients with CRC were extracted from GSE39582 in order to match the pathological characteristics of each group (Table 1). In GSE50081, patients with stage I-II LUAD were divided into two groups based on the risk value (Fig. 3G). The 1-years AUC value of the three-ISG-based signature supported the previous conclusion that the model had excellent accuracy and sensitivity in predicting LUAD patients' OS (Fig 3H). OS of patients in the low-risk group seemed obviously better than the high-risk group according to K-M survival analyses (Fig 3I). In GSE39582, patients with stage I-II CRC showed a similar distribution (Fig 3J). Although the predicting performance appeared to decline (Fig. 3K), the K-M survival analyses revealed that groups divided by three-ISG-based signature had a substantial discrepancy in OS (Fig. 3L).

By dividing patients in the TCGA-LUAD, GSE30219, GSE50081, and GSE39582 cohorts into subgroups according to their clinicopathological features, subgroup K-M survival analysis indicated that the signature had more exactly clinical values than distant metastasis for TCGA-LUAD patients (Fig. S3A-S3F). Whether gene signatures had the comparative predictive performance in patients with different tumor stages had contradictory conclusions (Fig. S3A, S3B, and S3G-S3J). In light of lymph nodes metastasis (LNM), the signature had more robust discrimination for LNM-negative LUAD patients in GSE50081 (Fig. S3K and S3L). Thus, the study included non-metastasis CRC patients from GSE39582 and proved that the signature better distinguished patients with different prognoses than other clinicopathological features (Table 2). This research team proved that signature had good differentiation performance in early, non-metastatic tumors.

**Figure 4:**
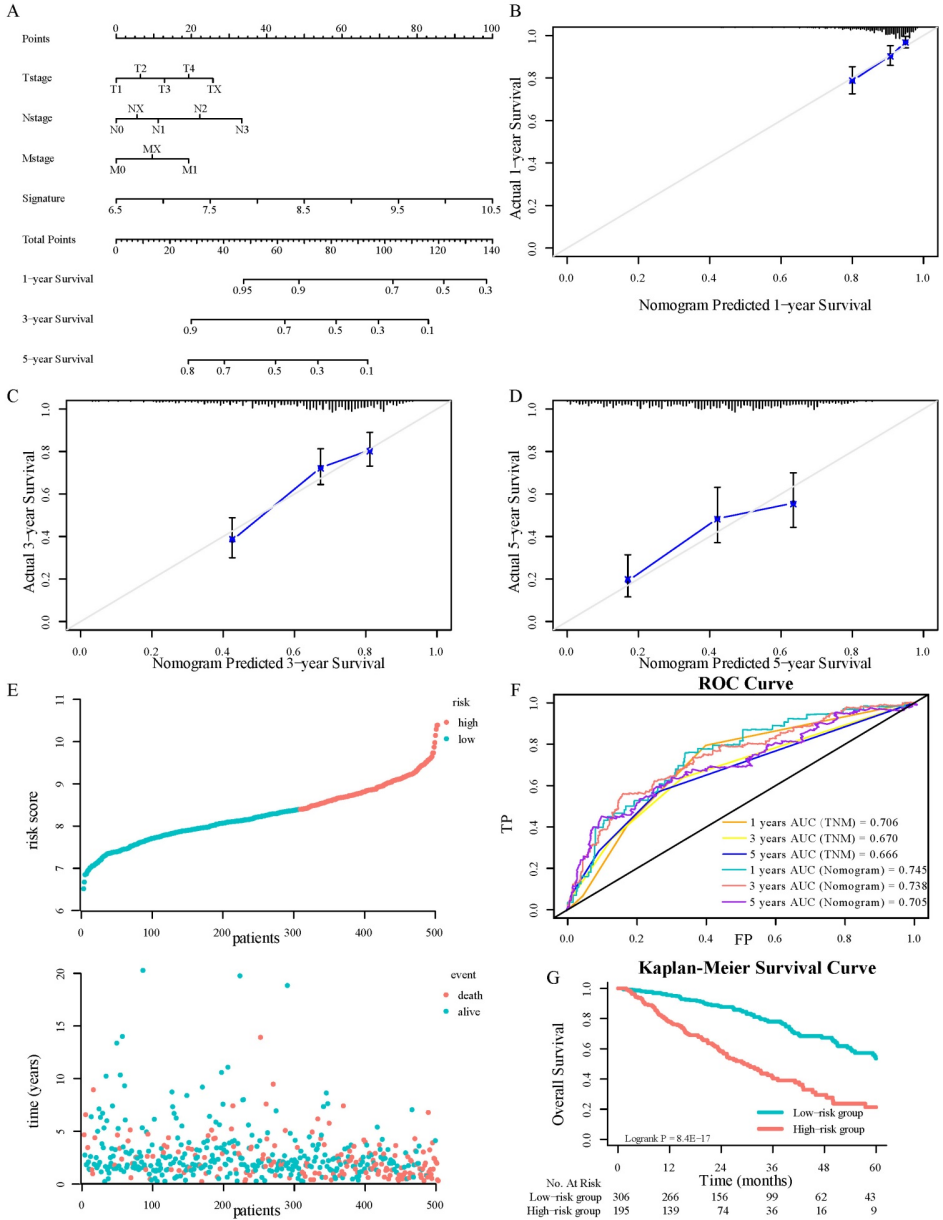
Nomograms convey the results of prognostic models using the 3-ISGs signature and TNM staging system to predict OS of patients with LUAD (A). The calibration curve for predicting patients' OS at 1-year(B). The calibration curve for predicting patients' OS l at 3-year (C). The calibration curve for predicting patients' OS at 5-year (D). The distributions of the risk score calculated by the nomogram and survival status of TCGA-LUAD patients (E). Time-dependent ROC analysis to assess the predictive function of the nomogram and TNM staging system at 1, 3, and 5 years (F). Kaplan-Meier survival curves of OS between high-risk and low-risk patients in the TCGA-LUAD cohort (G).

Comprehensively combining the results of univariable cox regression, four variables, including the signature, T stage, N stage, and M stage, were determined as independent predictive parameters to develop a nomogram in TCGA-LUAD cohorts (Figure 4A). The calibration curves were used to evaluate the reliability of nomogram prediction ability inconsistency with the standard curve (Fig. 4B-4D). The distributions of patients' survival and risk score calculated based on the nomogram were shown in Figure 4E. Investigators adapted the time-dependent ROC analyses to assess the predictive function of the nomogram, with AUC 0.745, 0.738, and 0.705 at 1-, 3-, and 5-years OS, respectively (Fig. 4F). Besides, the TNM staging system was crucial in clinical. To compare the ISGs signature with it, this research tested the AUC of the TNM stage and the signature by the bootstrap method (Figure 2G and 4F, *P* = 0.44), and there was no significant difference in sensitivity and specificity between the two prediction models, which demonstrated that the gene signature had the same predictive performance compared with TNM staging system. The K-M survival analyses displayed that the OS in the high-risk group was significantly worse (Fig. 4G, *P* < 0.0001).

### Potential biological function of the signature on tumor biology and microenvironment

**Figure 5:**
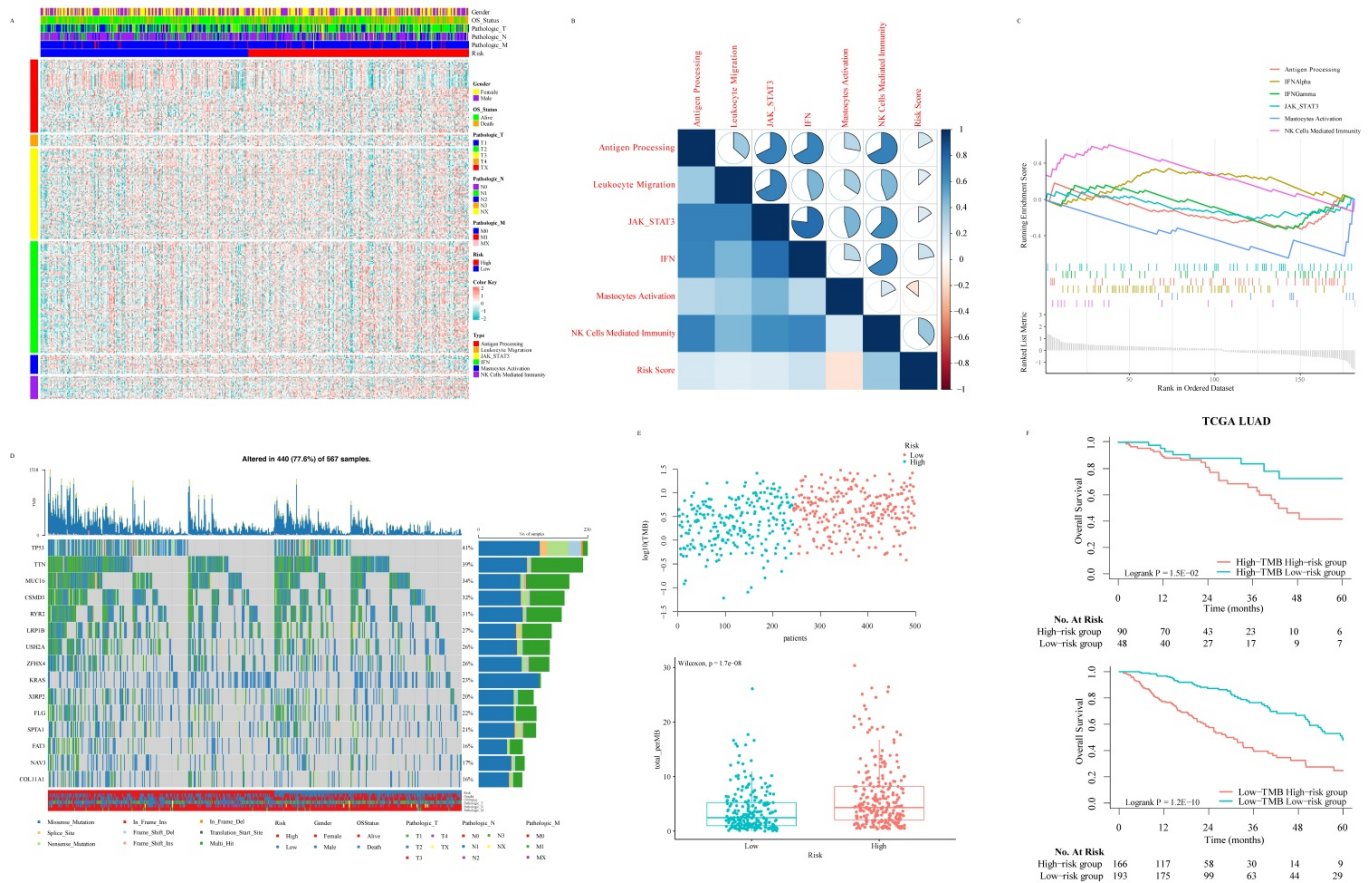
Relationship between risk score and immune-related signaling pathway in LUAD: The relationship between risk score and immune-related metagenes (A). Map of high-frequency mutation characteristics in patients from TCGA (B). The correlation analysis between risk score and metagenes (C). Significant gene sets enriched based on DEGs from LUAD patients with high- or low-risk scores (D). The distribution of TMB and risk scores (E). The survival analysis for LUAD patients according to risk score and TMB (F).

With the purpose of further gaining insight into the immunogenicity and microenvironment characteristics of ISG signature, we explored the relationship between the risk score and immune metagenes (Fig. 5A). The results showed that the risk score was not significantly correlated with the genomic changes of immune metagene (Fig. S4A) but significantly associated with natural killer (NK) cell activation at the transcriptome level (Fig. 5B and 5C), which put forward the potential mechanism of the ISG signature to predict the prognosis of LUAD patients. In order to study the relationship between TMB and ISG based signatures and verify signatures as potential markers of LUAD treatment response, we explored the distribution of TMB in ISG based signatures (Fig. 5D and 5E). TMB was positively correlated with the risk score, and the OS of LUAD patients with higher risk scores was worse than those with lower risk scores, no matter whether the patient's TMB was low or high (Fig. 5F).

**Figure 6:**
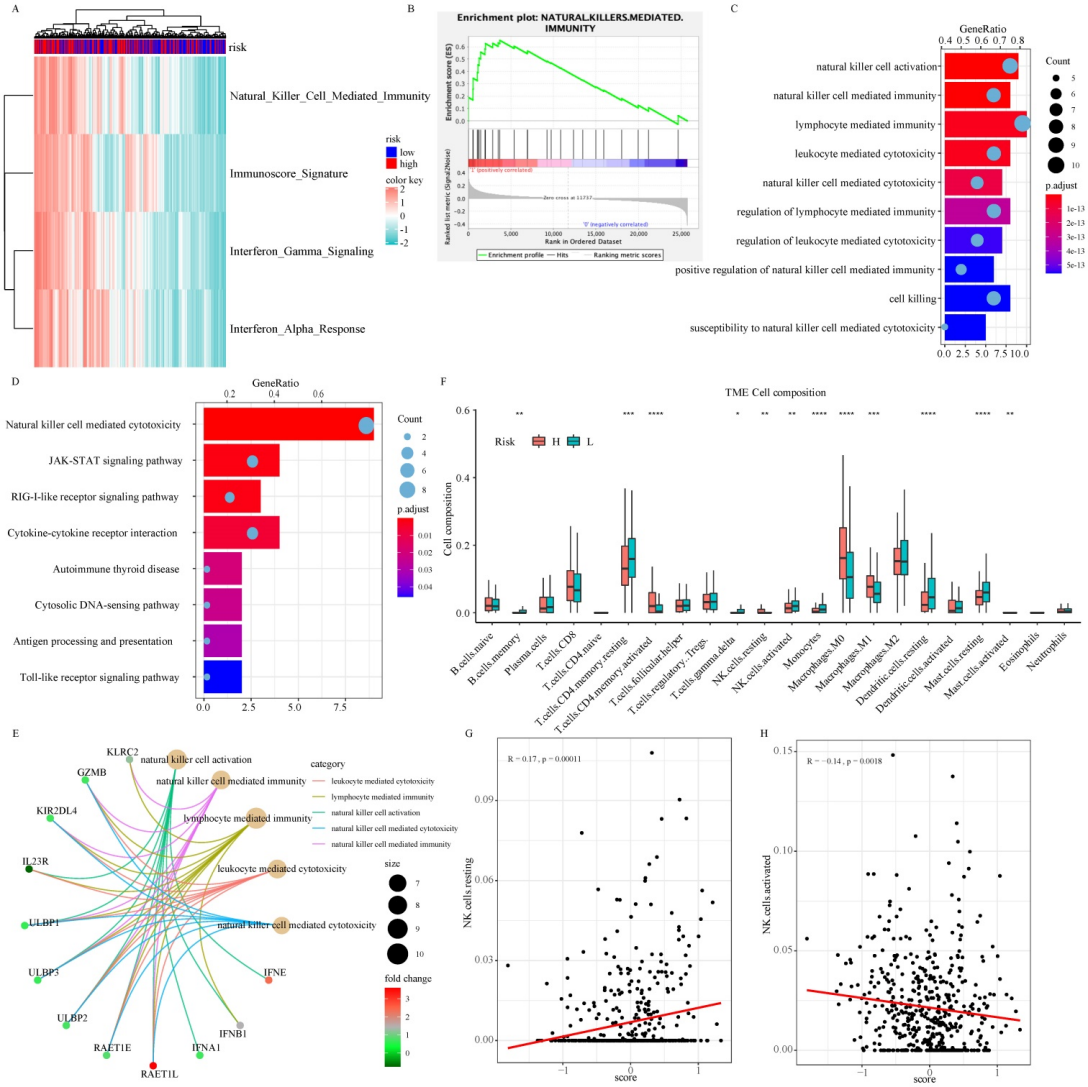
The biological function of the signature on tumor biology and microenvironment: The result of GSVA concentrating on immune-related signaling pathway (A). GSEA validated enhanced activity of natural killer cell mediated immunity pathway (B). GO (C and E) and KEGG (D) analysis of DEGs between high-risk and low-risk groups. The comparison of 22 immune cells infiltration levels in high- and low-risk groups in the TCGA cohort (F). The correlation analysis between resting (G) and activation (H) natural killer cell infiltration level and risk score.

As part of the ISG family, overexpression of NDC80, NPAS2, and AHNAK2 changed tumor microenvironment. In particular, they resulted in a decrease in the proportion of resting dendritic cells (DCs) and mast cells, an increase in the proportion of macrophage M0 and activation of CD4 memory T cell in the TCGA (Fig. 6F), GSE30219 (Fig. S4B-S4E), and GSE50081 (Fig S4F-S4I) cohorts. As shown in Figure 6A and 6B, there was a significant difference in NK cell mediated immunity pathway variation and enrichment score between patients in high-risk and low-risk groups according to the result of GSVA and GSEA. According to functional enrichment analysis of GO and KEGG pathway (Fig. 6C-6E), immunity and cytotoxicity mediated by NK cell activation were key biology processes for the formation of low-risk groups through JAK-STAT signaling pathway and cytokine-cytokine receptor interaction pathway. ULBP/RAET encoded ligands that affected the NK cells activation in downstream of the pathway. ULBP2 was highly expressed in patients in the high-risk group, which seemly was the reason for the increase of resting NK cells (Fig. 6E and Fig. S5A-S5B). Among all 22 immune cell types, resting NK cells were significantly positively correlated with signature while activated NK cells showed negative relation, indicating that the 3-ISGs signature was different from the previously concerned family member, CCDC92. This signature played a pivotal role in the immune regulation of cancer patients by affecting NK cells (Fig. 6F-6H). Similar results were obtained from the analysis of GSE30219 (Fig. S5C and S5D). In addition, biological function enrichment analysis indicated that chromosome segregation and proliferation-related pathways also made great contributions to the prognosis of LUAD patients (Fig. S5E).

### Verification of prognostic performance and biological function of ISG signature

**Figure 7:**
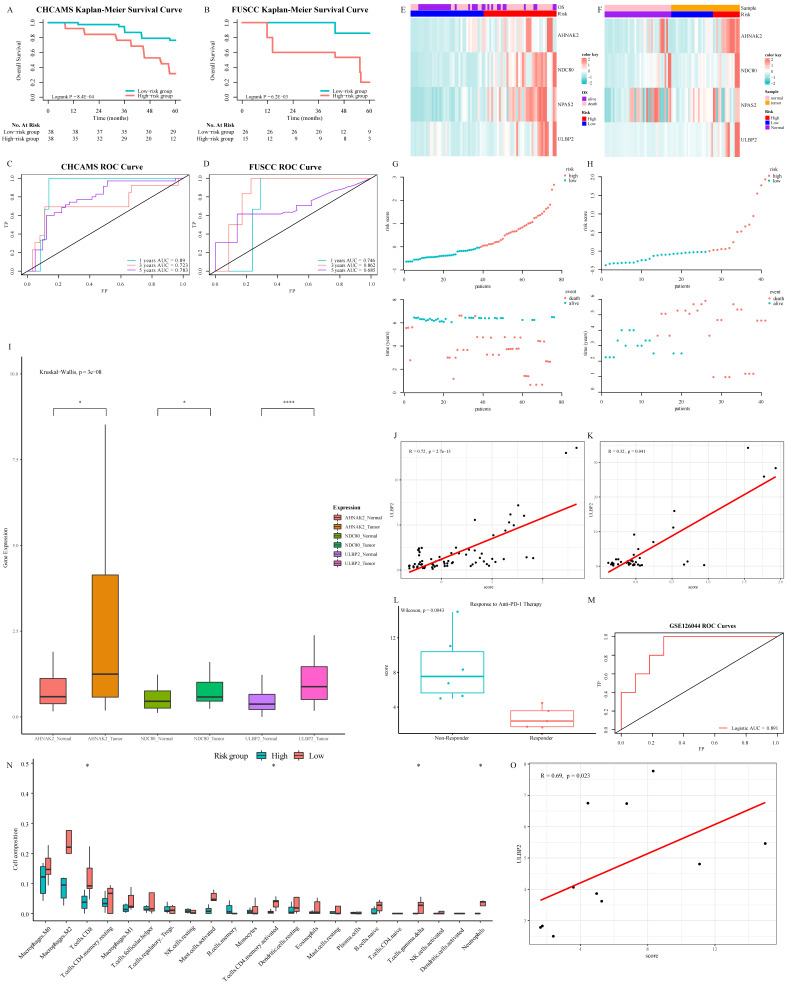
Verification of prognostic performance and biological function of 3-ISGs signature: Kaplan-Meier survival curves of OS between high-risk and low-risk patients in CHCAMS (A) and FUSCC (B) cohorts. Time-dependent ROC analyses in CHCAMS (C) and FUSCC (D) cohorts. The expression pattern of the 3-ISGs signature and clinical features of patients from CHCAMS (E) and FUSCC (F). The distributions of the risk score and survival status of CHCAMS (G) and FUSCC (H) patients. The different expression of differential ISGs and ULBP2 between tumor and non-tumor tissue in the FUSCC cohort (I). The correlation between risk score and ULBP2 in CHCAMS (J) and FUSCC (K) cohorts. The risk score between responder patients and non-responder patients during anti-PD-1 treatment (L). Logistic ROC analysis in GSE126044 (M). The comparison of 22 immune cells infiltration levels in high- and low-risk groups in the GSE120644 cohort (N). The correlation between risk score and ULBP2 in the GSE120644 cohort (O).

In the pan-cancer series, this signature had a valuable performance in distinguishing BCRA patients whose survival varied widely (Fig. S6A, S6C, and S6E). In consideration of KIRC, stage IV patients with better prognosis, who might benefit from nivolumab treatment, could be tagged by this signature (Fig. S6B, S6D, and S6F). In CHCAMS and FUSCC cohorts, based on individual expression of the 3 ISGs genes analyzed by Real-time PCR, we calculated the risk score in conformity with the same formula. Apparently, the survival time of the high-risk group was significantly shorter than that of the low-risk group (*P* < 0.05, Fig. 7A and 7B). The AUC was 0.89/0.746, 0.723/0.862, and 0.783/0.685 at 1, 3, and 5 years in CHCAMS/FUSCC cohorts, respectively (Fig. 7C and 7D). The expression pattern of 3-ISGs biomarkers and distribution of the 3 ISGs expression levels among pan-cancer patients belonging to the CHCAMS and FUSCC cohorts were shown in Figures 7E-7H. Besides, we analyzed the different expression levels of ISGs and ULBP2, a classical legend of NK cells, between tumor and non-tumor tissue in CRC cohorts (Fig. 7I). Considering the discrepancy between cancer and normal adjacent tissues, all data were acceptable, and except for NPAS2, the others had significant differences. Moreover, pan-cancer patients from the high-risk group apparently had high expression of NK cell ligands, such as ULBP2 (Fig. 7J, 7K, S6G and S6H). The Cox proportional hazards regression analysis was used to demonstrate the most significantly increased risk associated with signature in the CHCAMS and FUSCC cohorts (Table S4). The multivariate Cox regression in the training and validation cohort indicated that risk signature was the most stable evaluation variable, and the HR and 95% confidence interval (CI) were shown in Table 3. The detail of prognostic effects of time-dependent ROC analysis on the signature, TNM staging system, and nomogram in training and validation cohorts was displayed in Table S5. It was found that signature and TNM staging had considerable prognostic prediction performance, while the nomogram established by them performed best. In the post-immunotherapy cohort, the risk scores calculated using the same formula differed significantly between responders and non-responders during anti-PD-1 therapy (Fig.7L, *P* < 0.05). The AUC of the signature predicting response to anti-PD-1 therapy was 0.891 (Fig.7M, 95% CI 0.74-1.00), which signified that this gene signature can effectively predict the efficacy of immunotherapy. Because the efficacy of immunotherapy was closely related to T cells, the absolute value of tumor immune cell infiltration was calculated by the CIBERSORT algorithm (Fig. 7N). It was observed that the activated NK cells in the low-risk group were more than those in the high-risk group, despite the difference not being statistically significant. The strong correlation between the ISGs signature and the classical legend of NK cells, ULBP2, was demonstrated in the GSE120644 cohort (Fig. 7O). Besides, the levels of gamma delta T cells were significantly increased in the low-risk group, which partially confirmed the possible mechanism of the NK cell activation pathway. Comprehensively taking the result of the post-immunotherapy cohort into consideration, the ISG-related signature was expected to predict the response to immunotherapy, especially the treatment related to cytotoxic T cells and NK cells in the tumor microenvironment.

## Discussion

Due to inter-/intra-tumor heterogeneity, the prognosis of pan-cancer patients with the same pathological stage was of a wide variety, even among patients with localized carcinoma. In this study, three-ISGs signature, which was applied to evaluate OS in pan-cancer patients, and TNM staging system were incorporated to build a nomogram. The ROC and K-M survival curves indicated that the three-ISGs signature had robust discrimination performance. The application of the nomogram was confirmed regarding its calibration curve, which showed the possibility of OS predicted by the nomogram was highly consistent with the reality. Moreover, the obstruction of the NK cell activation pathway was considered as a cause of poor prognosis in biological function and tumor immune infiltration analysis.

Previous studies have tried to identify molecular markers for the detection of response to immunotherapy and OS in pan-cancer patients, especially in KIRC and LUAD 25, 26. Many reliable results have been achieved. Actually, programmed death ligand-1 tested by IHC was a predictive marker for immunotherapy in solid tumors 27. However, it did not have a direct correlation with the prognosis of patients, while the specificity of prediction could not reach the expectation. Because of durable survival benefit, PD-1/PD-L1 axis immunotherapy was the option of first-line treatment for LUAD patients 28. The efficacy of immunotherapy in patients with CRC or BRCA still needed more evidence to substantiate the application. Although immunotherapy prolonged patient survival in the clinic, the response rate of unselected pan-cancer patients was approximately 20%, observing acquired resistance 29, 30. Because only a minority of the patients was responsive to immunotherapy, efforts were made to determine the most suitable population for immunotherapy. Most studies concentrated on the evaluation of immune cells or tumor mutational burden on the efficacy of immunotherapy in pan-cancer patients, lack of evidence supporting at the transcriptional level 15, 31. Therefore, the 3-ISGs signature developed by us satisfied clinical demand, and efficiently and economically prognosticated the OS of pan-cancer patients.

Previous studies have demonstrated that ISGs were closely related to the innate immune killing of tumor malignant tumors 32. Hence, we focused on the prognosis of pan-cancer patients and ISGs expression profiles to find potential biomarkers. In our research, NDC80, NPAS2, and AHNAK2 were sifted out from the ISGs family to develop a signature that improved the prediction of OS for pan-cancer patients. The complex comprising of NDC80 protein located in the outer layer of the kinetochore and played a critical role in mitosis 33. Up-regulating expression level of NDC80 was negatively correlated with OS and positively correlated with immune infiltration cells, including regulatory T cells (Treg) and macrophages M0 in patients with hepatocellular carcinoma 34, which was consistent with the results of this study. Heretofore, none of studies has focused on how the up-regulated NDC80 in tumor tissue specifically affected the activation and biological properties of immune cells. NPAS2, the largest of the circadian genes, has been involved in tumorigenesis by forming heterodimers with BMAL1, which target the E-box sequence (CACGTG) to mediate the promoter of the oncogene c-myc and suppress its transcription^35^, ^36^. High expression of NPAS2 was correlated with poor survival in LUAD and liver cancer, and abnormal expression also contributed to T cell exhaustion and upregulation of immune checkpoint molecules and TNF signaling pathway 37, 38. Taking previous research and our results into consideration, overexpression of NPAS2 may negatively regulate DCs and positively associate macrophages. AHNAK2, a large nucleoprotein, was reported to be involved in the stress-induced non-classical FGF1 secretion pathway and shortened survival in patients with KIRC and thyroid cancer 39, 40. Besides, up-regulating expression of AHNAK2 was connected with lower activated NK cells and higher CD4 memory resting T cells and macrophages M0 infiltration, which was similar to the result of this research. Composing the signature, all three ISGs, NDC80, NPAS2, and AHNAK2, made contributions to poor OS and the suppressive immune microenvironment in general. Some NKG2D ligands, for example, ULBP/RAET family, were induced by overexpression of three ISGs and correlated with the prognosis of CRC patients 41.

Obviously, this research still has some limitations. First, this was a retrospective study that introduced inherent selection bias. Second, how interferon stimulated NDC80, NPAS2, and AHNAK2 expression still needs further cytology research. In addition, the model needs further optimization and prospective assessment to prove its clinical effectiveness.

## Conclusion

In conclusion, a three-ISGs signature was developed and validated for predicting the OS of pan-cancer patients. On account of this signature, a novel nomogram had sufficient discrimination and calibration capabilities and could be a handy instrument to decide patients' treatment strategies.

## Supplementary Material

Supplementary figures and tables 1, 4, 5.

Supplementary table 2.

Supplementary table 3.

## Figures and Tables

**Table 1:** Clinicopathological characteristics of patients in the training and validation cohorts (N (%)).

Characteristics	Training cohort			Validation cohort			
	TCGA-LUAD		GSE30219	GSE50081	GSE39582	CHCAMS	FUSCC
			N=83	N=127	N=229	N=76	N=41
Age							
<60	136 (27.1)		37 (44.6)	19 (15.0)	45 (19.7)	34 (44.7)	10 (24.4)
≥60	355 (70.9)		46 (55.4)	108 (85.0)	183 (79.9)	42 (55.3)	31 (75.6)
NA	10 (2.0)		0 (0)	0 (0)	1 (0.4)	0 (0)	0 (0)
Median (IQR)	66 (59-72)		60 (55-69)	69.9 (62.7-75.7)	69 (62-77)	60 (51-69)	63 (57-71)
Gender							
Female	268 (53.5)		18 (21.7)	62 (48.8)	92 (40.2)	48 (63.2)	14 (34.1)
Male	233 (46.5)		65 (78.3)	65 (51.2)	137 (59.8)	28 (36.8)	27 (65.9)
Primary site							
Right	298 (59.5)		\	\	87 (38.0)	0 (0)	20 (48.8)
Left	195 (38.9)		\	\	142 (62.0)	0 (0)	21 (51.2)
NA	8 (1.6)		83 (100)	127 (100)	0 (0)	76 (100)	0 (0)
T stage							
T0 and T1	166 (33.1)		69 (83.1)	43 (33.9)	9 (3.9)	6 (7.9)	0 (0)
T2	270 (53.9)		12 (14.5)	82 (64.5)	28 (12.2)	54 (71.1)	0 (0)
T3	44 (8.8)		2 (2.4)	2 (1.6)	192 (83.9)	7 (9.2)	11 (26.8)
T4	18 (3.6)		0 (0)	0 (0)	0 (0)	9 (11.8)	30 (73.2)
TX	3 (0.6)		0 (0)	0 (0)	0 (0)	0 (0)	0 (0)
N stage							
N0	326 (65.1)		80 (96.4)	94 (74.0)	229 (100)	35 (46.1)	41 (100)
N1	92 (18.3)		3 (3.6)	33 (26.0)	0 (0)	15 (19.7)	0 (0)
N2	68 (13.6)		0 (0)	0 (0)	0 (0)	26 (34.2)	0 (0)
N3	2 (0.4)		0 (0)	0 (0)	0 (0)	0 (0)	0 (0)
NX	13 (2.6)		0 (0)	0 (0)	0 (0)	0 (0)	0 (0)
M stage							
M0	474 (94.6)		83 (100)	127 (100)	229 (100)	76 (100)	41 (100)
M1	25 (5.0)		0 (0)	0 (0)	0 (0)	0 (0)	0 (0)
MX	2 (0.4)		0 (0)	0 (0)	0 (0)	0 (0)	0 (0)
OS							
Alive	320 (63.9)		40 (48.2)	76 (59.8)	175 (76.4)	35 (46.1)	15 (36.6)
Death	181 (36.1)		43 (51.8)	51 (40.2)	54 (23.5)	41 (53.9)	26 (63.4)
Median (IQR)	22.5 (14.5-37.8)		68.5 (28-113)	52.8 (24-69.6)	61 (37-85)	67.4 (44-76.6)	43.8 (30-63.2)
RFS							
Non-relapse	286 (57.1)		56 (67.5)	87 (68.5)	197 (86.0)	0 (0)	32 (78.0)
Relapse	215 (42.9)		27 (32.5)	37 (29.1)	32 (14.0)	0 (0)	9 (22.0)
NA	0 (0)		0 (0)	3 (2.4)	0 (0)	76 (100)	0 (0)
Median (IQR)	18.0 (10.1-29.6)		63 (18-107)	42 (15.6-64.8)	56 (30-83)	\	43.8 (12-60.8)

CHCAMS, Cancer Hospital Chinese Academy of Medical Sciences; FUSCC, Fudan University Shanghai Cancer Center; TCGA, The Cancer Genome Atlas; LUAD, lung adenocarcinoma; IQR, interquartile range; OS, Overall survival; RFS, Relapse-free survival

**Table 2:** Univariate Cox regression for overall survival in TCGA-LUAD, GSE30219, GSE50081 and GSE39582 cohorts.

Characteristics	TCGA-LUAD		GSE30219		GSE50081		GSE39582	
	HR (95% CI)	*P-*value	HR (95% CI)	*P-*value	HR (95% CI)	*P-*value	HR (95% CI)	*P-*value
Age		0.86		0.59		0.38		0.10
<60	1		1		1		1	
≥60	1.029 (0.742-1.428)		1.186 (0.636-2.211)		1.471 (0.626-3.457)		2.033 (0.868-4.762)	
Gender		0.59		0.78		0.23		0.12
Female	1		1		1		1	
Male	1.084 (0.809-1.452)		1.114 (0.516-2.403)		1.410 (0.807-2.463)		1.588 (0.892-2.828)	
Primary site		0.89		\		\		0.95
Left	1						1	
Right	1.022 (0.756-1.381)						1.017 (0.576-1.796)	
T stage		<0.001		0.1		0.004		0.9
T0 and T1	1		1		1		1	
T2	1.538 (1.072-2.206)	0.019	2.050 (1.022-4.114)	0.04	2.441 (1.217-4.896)	0.01	1.435 (0.168-12.30)	0.74
T3	2.969 (1.743-5.058)	<0.001	0.927 (0.125-6.889)	0.94	11.73 (2.502-54.99)	0.002	1.572 (0.217-11.41)	0.66
T4	3.129 (1.606-6.097)	<0.001						
TX	5.057 (1.214-21.074)	0.026						
N stage		<0.001		0.76		0.01		\
N0	1		1		1			
N1	2.454 (1.740-3.461)	<0.001	1.247 (0.299-5.202)		2.142 (1.199-3.825)			
N2	3.065 (2.086-4.504)	<0.001						
N3	Inf	0.99						
NX	1.411 (0.517-3.853)	0.50						
M stage		0.06		\		\		\
M0	1							
M1	2.275 (1.338-3.868)	0.002						
MX	3.127 (0.434-22.538)	0.2579						
Risk group		<0.001		0.001		0.025		0.022
Low risk	1		1		1		1	
High risk	2.553 (1.872-3.480)		2.741 (1.499-5.012)		1.917 (1.085-3.385)		1.903 (1.098-3.296)	

HR, hazard ratio; CI, confidence interval; TCGA, The Cancer Genome Atlas; LUAD, lung adenocarcinoma;

**Table 3:** Multivariate Cox regression for overall survival in training and validation cohorts.

Characteristics	TCGA-LUAD		GSE30219		GSE50081		GSE39582		CHCAMS		FUSCC		
	HR (95% CI)	*P*-value	HR (95% CI)	*P*-value	HR (95% CI)	*P*-value	HR (95% CI)	*P*-value	HR (95% CI)	*P*-value	HR (95% CI)	*P*-value	
Age		0.21		0.64		0.58		0.07		<0.01		0.20	
<60	1		1		1		1		1		1		
≥60	1.2 (0.9-1.8)		1.2 (0.6-2.2)		1.3 (0.5-3.1)		2.2 (0.9-5.3)		5.4 (2.3-13)		2.1 (0.7-6.7)		
Gender		0.93		0.79		0.15		0.08		0.02		0.54	
Female	1		1		1		1		1		1		
Male	1.0 (0.7-1.4)		0.9 (0.4-2.0)		1.5 (0.9-2.7)		1.7 (0.9-3.0)		0.4 (0.2-0.9)		0.7 (0.2-2.5)		
Primary site		0.98		\		\		0.78		\		<0.01	
Left	1						1				1		
Right	1.0 (0.7-1.4)						1.1 (0.6-1.9)				0.1 (0.03-0.4)		
T stage		0.09		0.52		0.02		0.68		0.92		0.22	
T0 and T1	1		1		1		1		1		\		
T2	1.2 (0.8-1.6)	0.64	1.5 (0.7-3.3)	0.28	1.9 (0.9-4.0)	0.08	1.2 (0.1-10)	0.87	0.9 (0.6-1.7)	0.92	\		
T3	2.4 (1.2-3.7)	<0.01	0.5 (0.1-6.5)	0.60	15 (2.3-91)	<0.01	1.4 (0.2-10)	0.76	2.0 (0.9-3.4)	0.90	1		
T4	3.5 (0.6-5.0)	0.40							2.9 (0.3-5.0)	0.99	2.0 (0.7-6.1)		
TX	4.7 (0.3-18)	0.42											
N stage		<0.01		0.81		0.03		\		0.02		\	
N0	1		1		1				1				
N1	2.1 (1.5-3.1)	<0.01	0.8 (0.1-4.9)		2.0 (1.1-3.6)				2.4 (1.1-6.0)	0.04			
N2	4.3 (3.0-6.7)	<0.01							0.4 (0.1-0.9)	0.03			
N3	Inf	0.99											
NX	1.1 (0.3-5.2)	0.77											
M stage		0.02		\		\		\		\		\	
M0	1												
M1	3.9 (2.1-7.5)	0.03											
MX	2.0 (0.4-2.6)	0.36											
Risk group		<0.01		<0.01		0.04		0.01		0.02		<0.01	
Low risk	1		1		1		1		1		1		
High risk	2.3 (1.7-3.2)		2.8 (1.4-5.3)		1.8 (1.1-3.2)		2.0 (1.2-3.5)		1.6 (1.1-2.5)		8.3 (2.9-24)		

TCGA, The Cancer Genome Atlas; LUAD, lung adenocarcinoma; CHCAMS, Cancer Hospital Chinese Academy of Medical Sciences; FUSCC, Fudan University Shanghai Cancer Center; HR, hazard ratio; CI, confidence interval

## Abbreviations

BRCA: breast cancer
CRC: colorectal cancer
LUAD: lung adenocarcinoma
COAD: colon adenocarcinoma
KIRC: kidney clear cell carcinoma
ISGs: interferon-stimulated genes
JAK-STAT1: Janus kinase-signal transducer and activator of transcription 1
OS: overall survival
K-M: Kaplan-Meier
LIMMA: linear model for microarray data
DEGs: differentially expressed genes
AUC: area under the curve
NCC: National Cancer Center
CHCAMS: Cancer Hospital, Chinese Academy of Medical Sciences
FUSCC: Fudan University Shanghai Cancer Center
GSVA: gene set variation analysis
GSEA: gene set enrichment analysis
RFS: relapse-free survival
LNM: lymph nodes metastasis
NK: natural killer
DCs: dendritic cells
CI: confidence interval
Treg: regulatory T cells
