# Supplementary material for: Establishment and Validation of an Interferon-Stimulated Genes (ISGs) Prognostic Signature in Pan-cancer Patients: A Multicenter, Real-world Study
Source: Int J Biol Sci. 2022 May 21;18(9):3762–76. doi: 10.7150/ijbs.71385 (PMC9254476; doi:10.7150/ijbs.71385)
Supplement: Supplementary file 1 — Supplementary figures and tables 1, 4, 5. [file ijbsv18p3762s1.pdf]

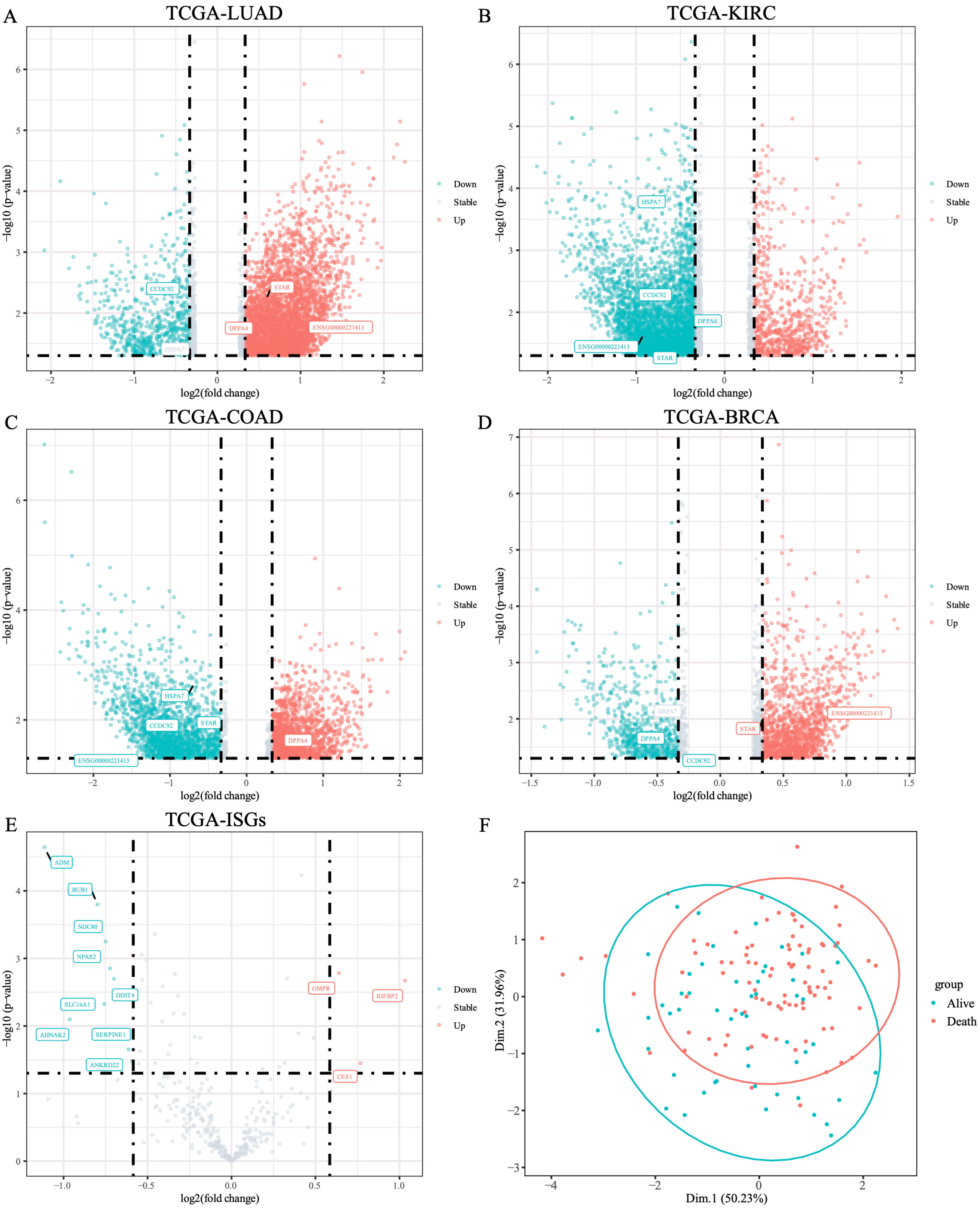

A
TCGA-LUAD
B
TCGA-KIRC
C
TCGA-COAD
D
TCGA-BRCA
E
TCGA-ISGs
F
−log10 (p−value)
log2(fold change)
Down
Stable
Up
CCDC92
STAR
DPPA4
ENSG00000221413
HSPA7
ADM
BUB1
NDC80
NPAS2
DDIT4
SLC16A1
AHNAK2
SERPINE1
ANKRD22
GMPR
IGFBP2
CES1
Dim.1 (50.23%)
Dim.2 (31.96%)
group
Alive
Death

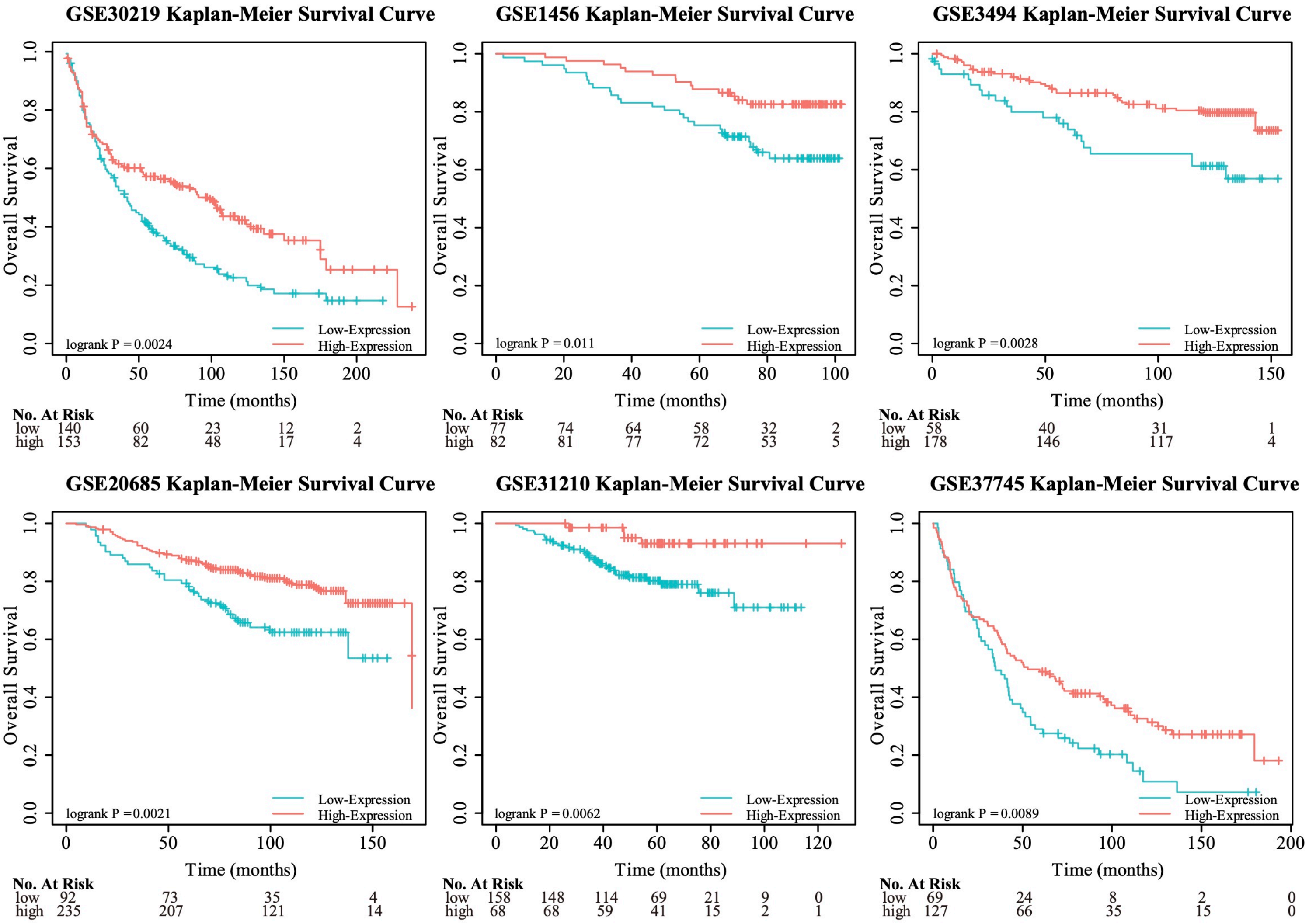
GSE30219 Kaplan-Meier Survival Curve
Overall Survival
Time (months)
logrank P =0.0024
Low-Expression
High-Expression
No. At Risk
low 140 60 23 12 2
high 153 82 48 17 4
GSE1456 Kaplan-Meier Survival Curve
Overall Survival
Time (months)
logrank P =0.011
Low-Expression
High-Expression
No. At Risk
low 77 74 64 58 32 2
high 82 81 77 72 53 5
GSE3494 Kaplan-Meier Survival Curve
Overall Survival
Time (months)
logrank P =0.0028
Low-Expression
High-Expression
No. At Risk
low 58 40 31 1
high 178 146 117 4
GSE20685 Kaplan-Meier Survival Curve
Overall Survival
Time (months)
logrank P =0.0021
Low-Expression
High-Expression
No. At Risk
low 92 73 35 4
high 235 207 121 14
GSE31210 Kaplan-Meier Survival Curve
Overall Survival
Time (months)
logrank P =0.0062
Low-Expression
High-Expression
No. At Risk
low 158 148 114 69 21 9 0
high 68 68 59 41 15 2 1
GSE37745 Kaplan-Meier Survival Curve
Overall Survival
Time (months)
logrank P =0.0089
Low-Expression
High-Expression
No. At Risk
low 69 24 8 2 0
high 127 66 35 15 0

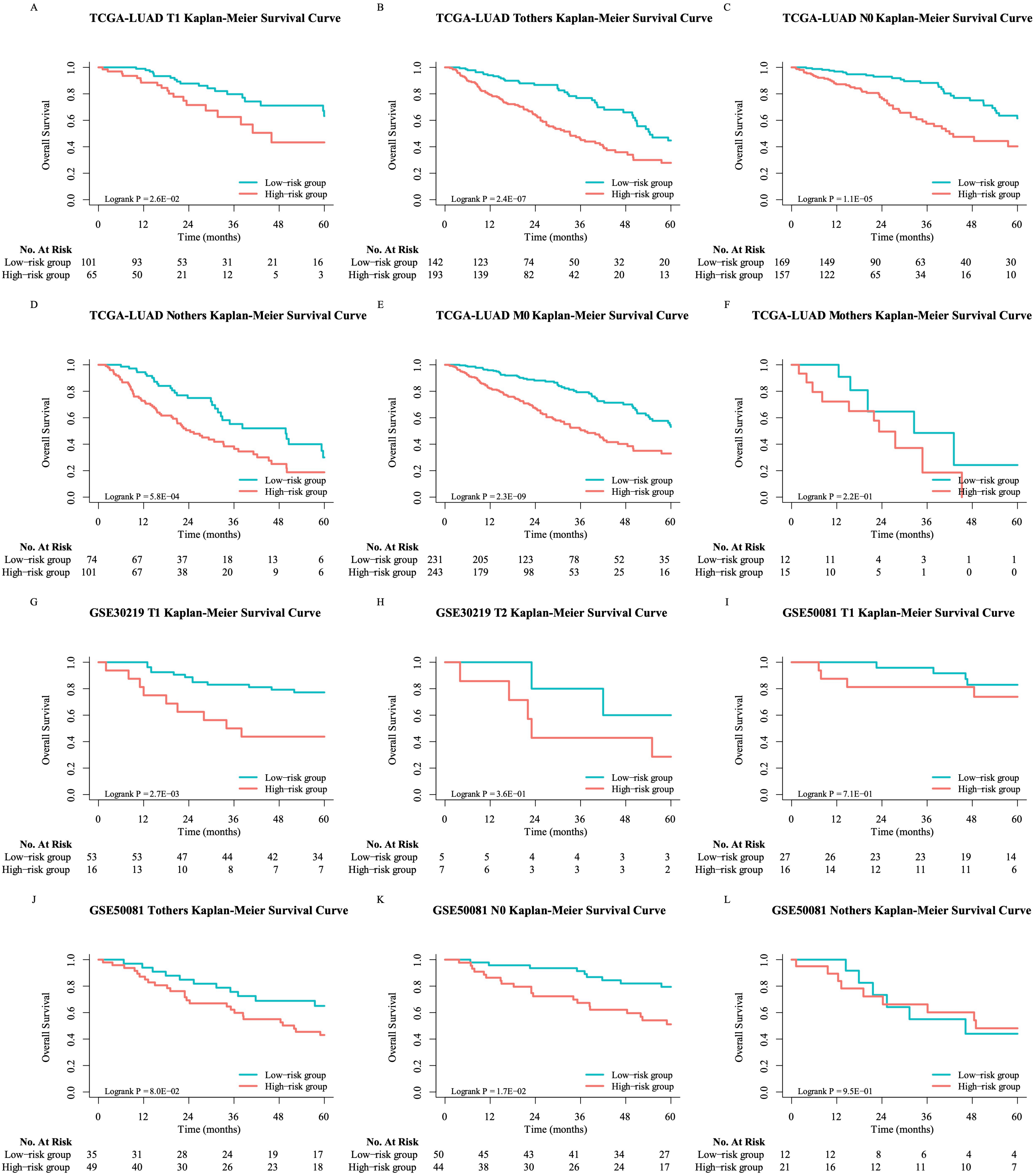
A
TCGA-LUAD T1 Kaplan-Meier Survival Curve
Overall Survival
Time (months)
Low−risk group
High−risk group
Logrank P = 2.6E−02
No. At Risk
Low−risk group 101 93 53 31 21 16
High−risk group 65 50 21 12 5 3
B
TCGA-LUAD Tothers Kaplan-Meier Survival Curve
Logrank P = 2.4E−07
No. At Risk
Low−risk group 142 123 74 50 32 20
High−risk group 193 139 82 42 20 13
C
TCGA-LUAD N0 Kaplan-Meier Survival Curve
Logrank P = 1.1E−05
No. At Risk
Low−risk group 169 149 90 63 40 30
High−risk group 157 122 65 34 16 10
D
TCGA-LUAD Nothers Kaplan-Meier Survival Curve
Logrank P = 5.8E−04
No. At Risk
Low−risk group 74 67 37 18 13 6
High−risk group 101 67 38 20 9 6
E
TCGA-LUAD M0 Kaplan-Meier Survival Curve
Logrank P = 2.3E−09
No. At Risk
Low−risk group 231 205 123 78 52 35
High−risk group 243 179 98 53 25 16
F
TCGA-LUAD Mothers Kaplan-Meier Survival Curve
Logrank P = 2.2E−01
No. At Risk
Low−risk group 12 11 4 3 1 1
High−risk group 15 10 5 1 0 0
G
GSE30219 T1 Kaplan-Meier Survival Curve
Logrank P = 2.7E−03
No. At Risk
Low−risk group 53 53 47 44 42 34
High−risk group 16 13 10 8 7 7
H
GSE30219 T2 Kaplan-Meier Survival Curve
Logrank P = 3.6E−01
No. At Risk
Low−risk group 5 5 4 4 3 3
High−risk group 7 6 3 3 3 2
I
GSE50081 T1 Kaplan-Meier Survival Curve
Logrank P = 7.1E−01
No. At Risk
Low−risk group 27 26 23 23 19 14
High−risk group 16 14 12 11 11 6
J
GSE50081 Tothers Kaplan-Meier Survival Curve
Logrank P = 8.0E−02
No. At Risk
Low−risk group 35 31 28 24 19 17
High−risk group 49 40 30 26 23 18
K
GSE50081 N0 Kaplan-Meier Survival Curve
Logrank P = 1.7E−02
No. At Risk
Low−risk group 50 45 43 41 34 27
High−risk group 44 38 30 26 24 17
L
GSE50081 Nothers Kaplan-Meier Survival Curve
Logrank P = 9.5E−01
No. At Risk
Low−risk group 12 12 8 6 4 4
High−risk group 21 16 12 11 10 7

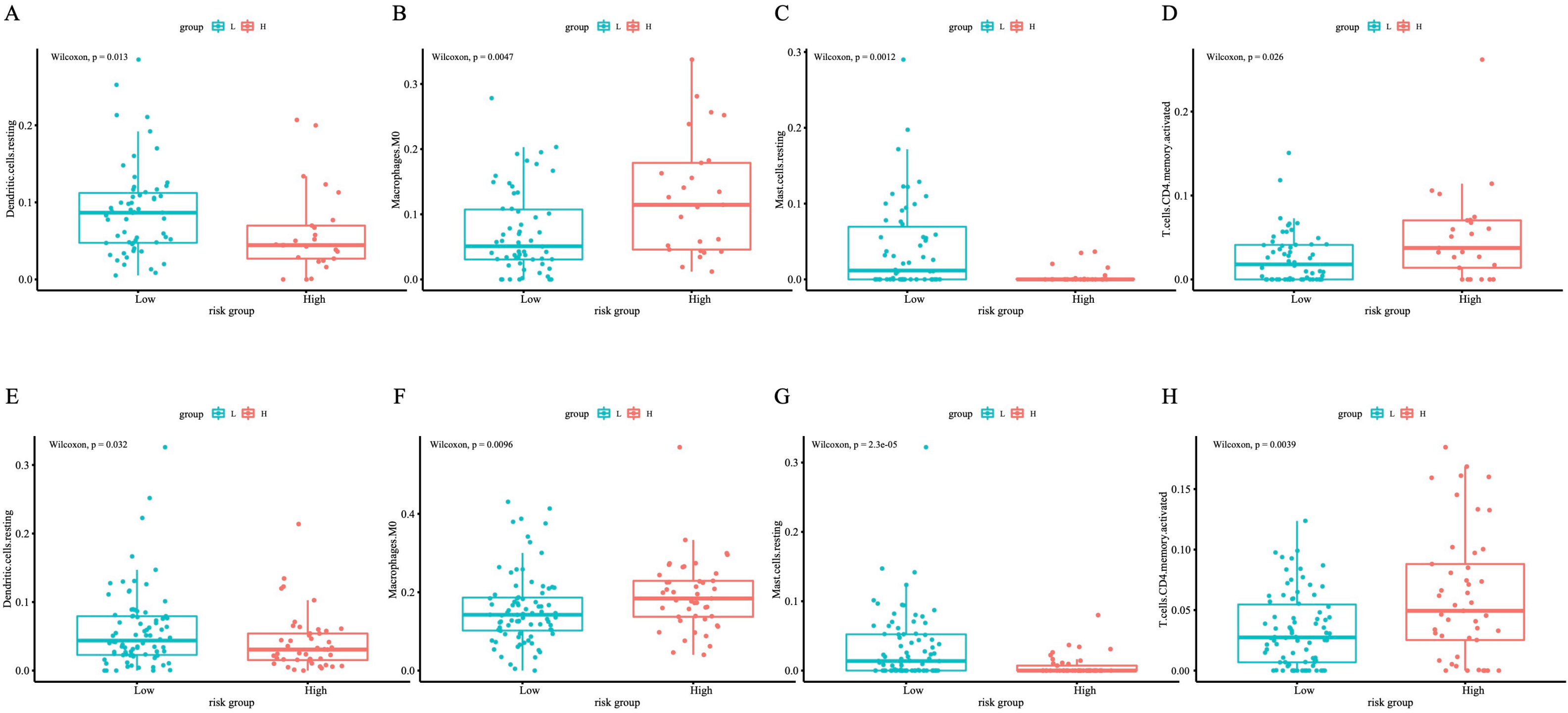
A
B
C
D
E
F
G
H
group L H
Wilcoxon, p = 0.013
Wilcoxon, p = 0.0047
Wilcoxon, p = 0.0012
Wilcoxon, p = 0.026
Wilcoxon, p = 0.032
Wilcoxon, p = 0.0096
Wilcoxon, p = 2.3e-05
Wilcoxon, p = 0.0039
Dendritic.cells.resting
Macrophages.M0
Mast.cells.resting
T.cells.CD4.memory.activated
Low
High
risk group

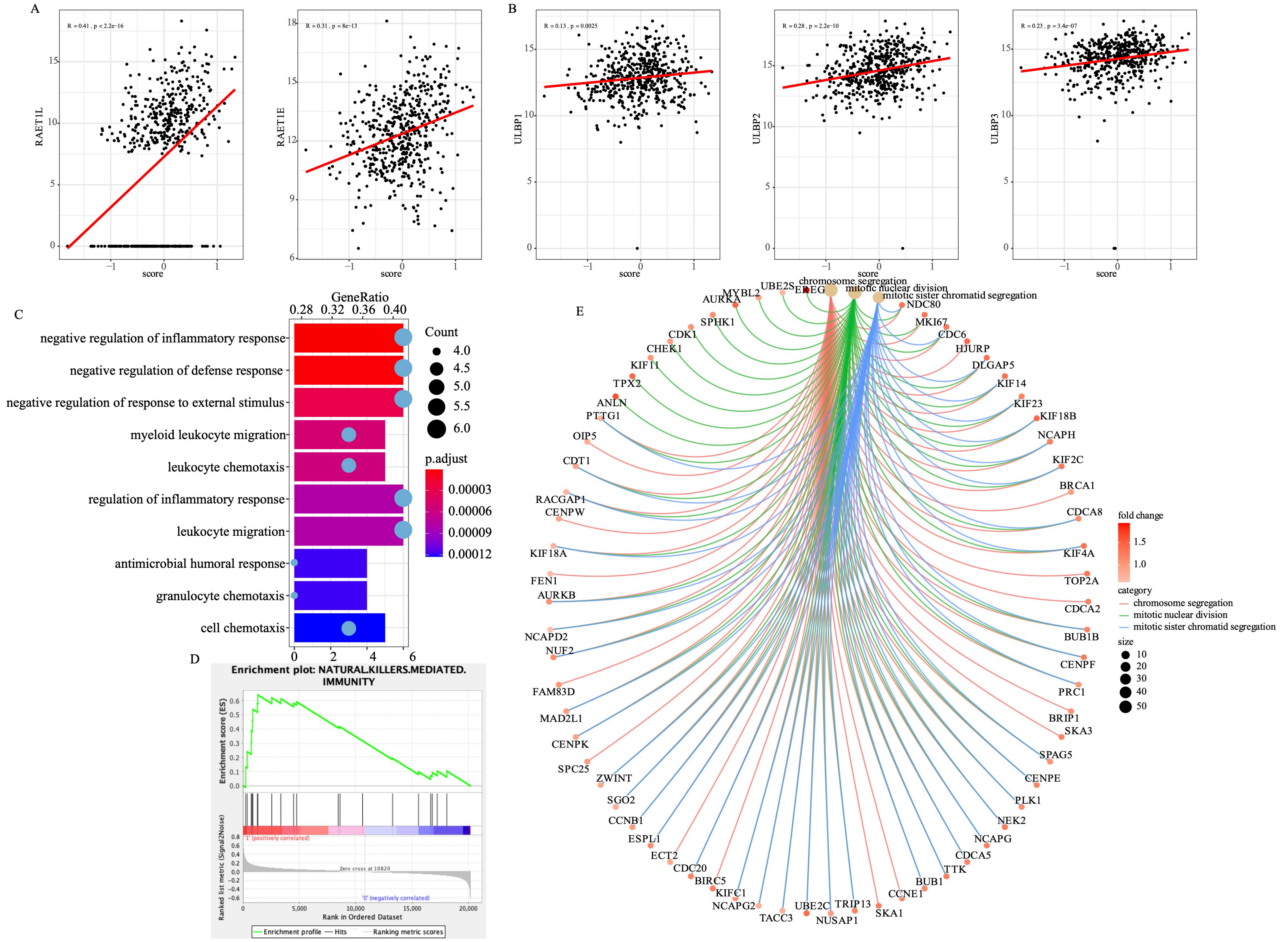
A
R = 0.41 , p < 2.2e−16
RAET1L
score
R = 0.31 , p = 8e−13
RAET1E
score
B
R = 0.13 , p = 0.0025
ULBP1
score
R = 0.28 , p = 2.2e−10
ULBP2
score
R = 0.23 , p = 3.4e−07
ULBP3
score
C
GeneRatio
0.28 0.32 0.36 0.40
negative regulation of inflammatory response
negative regulation of defense response
negative regulation of response to external stimulus
myeloid leukocyte migration
leukocyte chemotaxis
regulation of inflammatory response
leukocyte migration
antimicrobial humoral response
granulocyte chemotaxis
cell chemotaxis
0 2 4 6
Count
4.0
4.5
5.0
5.5
6.0
p.adjust
0.00003
0.00006
0.00009
0.00012
D
Enrichment plot: NATURAL.KILLERS.MEDIATED.IMMUNITY
Enrichment score (ES)
Ranked list metric (Signal2Noise)
'1' (positively correlated)
Zero cross at 10820
'0' (negatively correlated)
Rank in Ordered Dataset
Enrichment profile
Hits
Ranking metric scores
E
chromosome segregation
mitotic nuclear division
mitotic sister chromatid segregation
UBE2S
EREG
MYBL2
AURKA
SPHK1
CDK1
CHEK1
KIF11
TPX2
ANLN
PTTG1
OIP5
CDT1
RACGAP1
CENPW
KIF18A
FEN1
AURKB
NCAPD2
NUF2
FAM83D
MAD2L1
CENPK
SPC25
ZWINT
SGO2
CCNB1
ESPL1
ECT2
CDC20
BIRC5
KIFC1
NCAPG2
TACC3
UBE2C
NUSAP1
TRIP13
SKA1
CCNE1
BUB1
TTK
CDCA5
NCAPG
NEK2
PLK1
CENPE
SPAG5
SKA3
BRIP1
PRC1
CENPF
BUB1B
CDCA2
TOP2A
KIF4A
CDCA8
BRCA1
KIF2C
NCAPH
KIF18B
KIF23
KIF14
DLGAP5
HJURP
CDC6
MKI67
NDC80
fold change
1.5
1.0
category
chromosome segregation
mitotic nuclear division
mitotic sister chromatid segregation
size
10
20
30
40
50

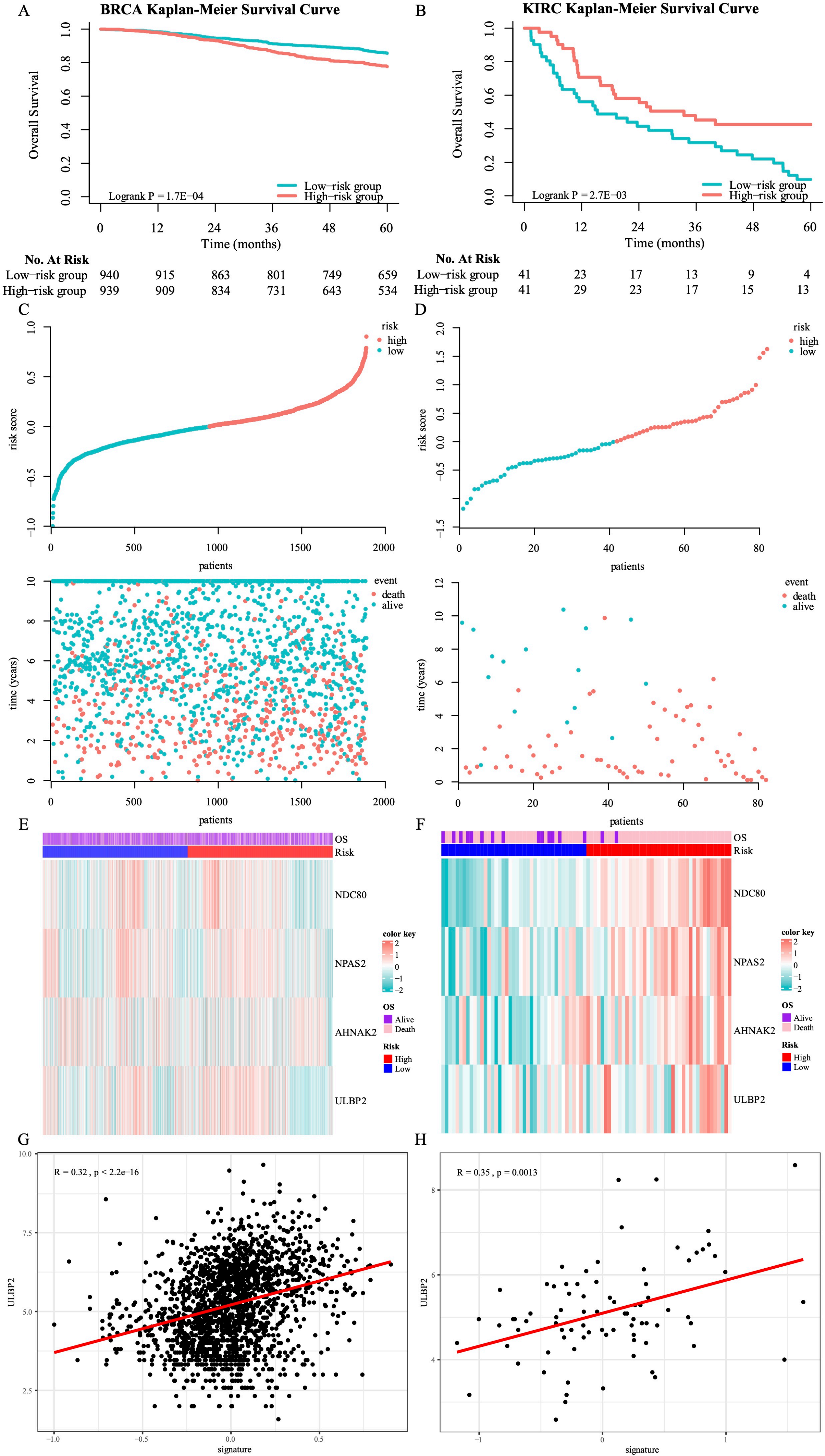
A
BRCA Kaplan-Meier Survival Curve
Overall Survival
Logrank P = 1.7E−04
Low−risk group
High−risk group
Time (months)
No. At Risk
Low−risk group 940 915 863 801 749 659
High−risk group 939 909 834 731 643 534
B
KIRC Kaplan-Meier Survival Curve
Overall Survival
Logrank P = 2.7E−03
Low−risk group
High−risk group
Time (months)
No. At Risk
Low−risk group 41 23 17 13 9 4
High−risk group 41 29 23 17 15 13
C
D
risk score
risk
high
low
patients
time (years)
event
death
alive
E
F
OS
Risk
NDC80
NPAS2
AHNAK2
ULBP2
color key
OS
Alive
Death
Risk
High
Low
G
R = 0.32 , p < 2.2e−16
ULBP2
signature
H
R = 0.35 , p = 0.0013
ULBP2
signature

Supplementary Table 1 The primers used to amplify specific genes in CHCAMS and FUSCC cohorts

Gene symbol	Primer name	Sequence
NDC80	NDC80 F	TTC AGT TTC CAG CGG TGG TG
	NDC80 R	TCC GGG ATC CAT GTC CAC TA
NPAS2	NPAS2 F	CGT GTT GGA AAA GGT CAT CGG
	NPAS2 R	TCC AGT CTT GCT GAA TGT CAC
AHNAK2	AHNAK2 F	GTG CAG AAA CGG AAG ATG ACC
	AHNAK2 R	GCC TCA GTC GTG TAT TCG TAG A
ULBP2	ULBP2 F	AGC AAC TGC GTG ACA TTC AG
	ULBP2 R	GCC ATC CTA TAC AGT CTC CCA

CHCAMS, Cancer Hospital Chinese Academy of Medical Sciences; FUSCC, Fudan University Shanghai Cancer Center; NDC80, NDC80 kinetochore complex component; NPAS2, neuronal PAS domain protein 2; AHNAK2, AHNAK nucleoprotein 2; ULBP2, UL16 binding protein 2;

Supplementary Table 4 Univariate Cox regression for overall survival in CHCAMS and FUSCC cohorts.

Characteristics	CHCAMS		FUSCC	
	HR (95% CI)	p value	HR (95% CI)	p value
Age		0.001		0.33
<60	1		1	
≥60	3.049 (1.569-5.923)		0.648 (0.272-1.548)	
Gender		0.004		0.57
Female	1		1	
Male	2.570 (1.356-4.873)		0.768 (0.310-1.900)	
Primary site		\		0.04
Left			1	
Right			0.349 (0.130-0.936)	
T stage		0.37		0.72
T0 and T1	1		\	
T2	1.191 (0.814-1.741)	0.37	\	
T3	2.382 (0.407-3.482)	0.36	1	
T4	3.573 (0.271-5.223)	0.40	1.165 (0.511-2.655)	
TX			\	
N stage		0.04		\
N0	1			
N1	1.740 (0.837-3.616)	0.13		
N2	0.546 (0.237-1.258)	0.16		
N3				
NX				
M stage		\		\
M0				
M1				
MX				
Risk group		<0.001		0.006
Low risk	1		1	
High risk	3.062 (1.536-6.104)		2.958 (1.312-6.669)	

CHCAMS, Cancer Hospital Chinese Academy of Medical Sciences; FUSCC, Fudan University Shanghai Cancer Center; HR, hazard ratio; CI, confidence interval;

Supplementary Table 5 Prediction performance of ROC analysis in the training and validation cohorts

Characteristics	TCGA-LUAD			Validation cohorts (signature)				
	signature	TNM system	nomogram	GSE30219	GSE50081	GSE39582	CHCAMS	FUSCC
C-index	0.646	0.666	0.705	0.796	0.648	0.642	0.783	0.685
95% CI	0.597-0.759	0.573-0.750	0.621-0.789	0.700-0.890	0.528-0.747	0.467-0.766	0.696-0.869	0.591-0.770
Sensitivity	0.835	0.794	0.758	0.824	0.874	0.838	0.600	0.615
Specificity	0.528	0.600	0.660	0.722	0.422	0.583	0.878	0.860
Best cutoff	8.716	\	62.03	3.163	2.583	3.420	1.722	0.644

ROC, receiver operating characteristic; TCGA, The Cancer Genome Atlas; LUAD, lung adenocarcinoma; TNM, tumor–node–metastasis; CHCAMS, Cancer Hospital Chinese Academy of Medical Sciences; FUSCC, Fudan University Shanghai Cancer Center; CI, confidence interval
